# Integrated Lab-on-a-Chip Optical Biosensor Using Ultrathin Silicon Waveguide SOI MMI Device †

**DOI:** 10.3390/s20174955

**Published:** 2020-09-01

**Authors:** Mohamed Y. Elsayed, Sherif M. Sherif, Amina S. Aljaber, Mohamed A. Swillam

**Affiliations:** 1 Institute of Biomedical Engineering (BME), University of Toronto, Toronto, ON M5S 3E2, Canada; mohammed.elsayed@mail.utoronto.ca; 2 Department of Chemistry and Earth Sciences, College of Arts and Sciences, Qatar University, Doha 2713, Qatar; sherifms@aucegypt.edu (S.M.S.); a.s.aljaber@qu.edu.qa (A.S.A.); 3 School of Science and Engineering, Department of Physics, The American University in Cairo, New Cairo 11835, Egypt

**Keywords:** optical biosensors, ultrathin silicon waveguides, multimode interference

## Abstract

Waveguides with sub-100 nm thickness offer a promising platform for sensors. We designed and analyzed multimode interference (MMI) devices using these ultrathin platforms for use as biosensors. To verify our design methodology, we compared the measured and simulated spectra of fabricated 220-nm-thick MMI devices. Designs of the MMI biosensors based on the sub-100 nm platforms have been optimized using finite difference time domain simulations. At a length of 4 mm, the 50-nm-thick MMI sensor provides a sensitivity of roughly 420 nm/RIU and with a figure of merit (FOM) definition of sensitivity/full-width-at-half-maximum, the FOM is 133. On the other hand, using a thickness of 70 nm results in a more compact design—only 2.4 mm length was required to achieve a similar FOM, 134, with a sensitivity of 330 nm/RIU. The limits of detection (LOD) were calculated to be 7.1 × 10^−6^ RIU and 8.6 × 10^−6^ RIU for the 50 nm and the 70-nm-thick sensor, respectively. The LOD for glucose sensing was calculated to be less than 10 mg dL^−1^ making it useful for detecting glucose in the diabetic range. The biosensor is also predicted to be able to detect layers of protein, such as biotin-streptavidin as thin as 1 nm. The ultrathin SOI waveguide platform is promising in biosensing applications using this simple MMI structure.

## 1. Introduction

Optical biosensors are becoming more important for lab-on-a-chip applications because they allow the analysis of a large variety of analytes and monitoring reactions in real time with high temporal resolution [1]. Optical biosensors based on refractive index change could be used as label-free detection methods for a variety of lab-on-a-chip applications.

The main challenge is that on-chip optical sensors tend to be difficult to integrate with other technologies. Ease of integration with microfluidic and electronic components is a significant feature for a biosensor to be considered a viable solution. In addition, strong light-matter interaction is an important parameter in sensors and has been achieved through resonance, slot waveguides, and plasmonics [1,2,3,4,5,6,7,8,9,10,11,12]. A simpler approach would be to use a waveguide platform that leaks a lot of the light into the surrounding material, such as vertical nanowire sensors [13,14] or suspended waveguides [15]. Thus, the performance of the waveguide will be strongly affected by the refractive index of the surrounding material and can be used as a sensor. However, such approaches require high-resolution lithography, which can complicate fabrication.

We started to explore the ultrathin waveguide platform and to assess its potential in sensing applications [16]. The ultrathin waveguide platform has many of the advantages of the more conventional (thicker) silicon platforms, such as possible integration with electronics, with the additional advantage of easy fabrication. The relatively larger waveguide of width larger than 1 μm, means that advanced lithographic techniques are not required [17]. Silicon nitride waveguides with 50 to 100 nm thickness were previously proposed [18], and ring resonators were designed using these waveguides [19,20]. The ultrathin silicon-on-insulator platform has been gaining popularity recently, and a variety of devices have been proposed. The first ultrathin silicon-on-insulator (SOI) waveguides were developed for on-chip optical networks, focusing on low losses, demonstrating a loss of 0.3 dB cm^−1^ [21]. Subsequently, the ultrathin SOI has started to gain popularity for the communications applications, focusing on low losses, and a variety of devices were developed, such as 50 nm thick mode couplers [22] and grating couplers [23], 60-nm-thick Bragg gratings, 1 × 2 multimode interference (MMI) couplers, and Mach-Zender interferometers (MZIs), [24], and a 90-nm thick microring-based laser [25]. Ninety-nm thick ring resonators were used as sensors, showing intrinsic limits of detection on the order of 5 × 10^−4^ [26]. For the silicon-ultrathin waveguide proposed in this work, grating couplers were previously designed to couple light to optical fibers with 50% efficiency [27].

The multimode interference device consists of three regions; the first region serves as the input, it is a single-mode waveguide for delivering the fundamental mode to the second region, which is a wide waveguide supporting multiple modes, the third region is the output single-mode waveguide. The multimode interference device relies on the self-imaging concept, where the input field is reconstructed in single or multiple images periodically along the propagation direction of the waveguide [28]. The multimode interference has been vastly employed in fibers for a variety of applications, including sensing [29]. On-chip MMI devices have been widely used for signal processing applications such as waveguide division multiplexing and power splitting [24,30]. A few on-chip MMI sensors were also proposed [31,32], including a silicon-based MMI temperature sensor [33]. After presenting the results, there will be a comparison between this work and state of the art MMI sensors. 

## 2. Methods

### 2.1. Simulation—Analysis and Design Methodology

**Figure 1:**
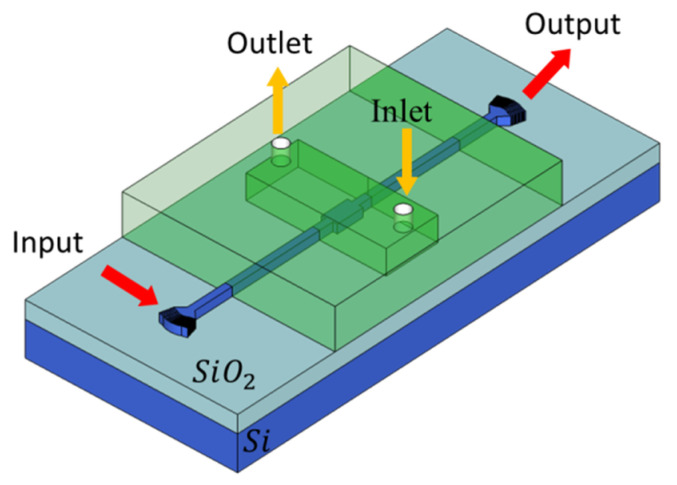
3D Schematic of MMI device based on an SOI wafer inside microfluidic channel (not to scale). Light is coupled by the use of grating couplers at the input and output. The microfluidic channel has an inlet and outlet for injecting analyte.

A 1.55 µm infrared laser source is used for excitation, which is widely used in biosensing and communication applications. Light is coupled to and out of the MMI device through fully-etched grating couplers. Light propagation, self-imaging, and spectral shifts are produced in the wider MMI section, which is a single input-single output MMI device with symmetric injection, as shown in Figure 1. The output spectrum is analyzed by an infrared semiconductor photodetector. The design is based on the SOI platform with a 2 μm thick buried oxide layer and varying thickness layers of the device layer.

Sensing is based on the self-imaging principle of multimode interferometers. The beating length is where interference of the first two fundamental modes is maximum and is given by (1):

(1) $$L_{\pi }=(4n_{eff}W_{e}^{2})/3\lambda$$

where *n_eff_* is the effective index of the *TE*_0_ mode, λ is the operating wavelength 1550 nm, *W_e_* is the effective width of the multimode section, which was taken as the geometrical width in this work. Thus, the refractive index of the superstrate (to be the analyte) affects the beating length through *n_eff_*.

**Figure 2:**
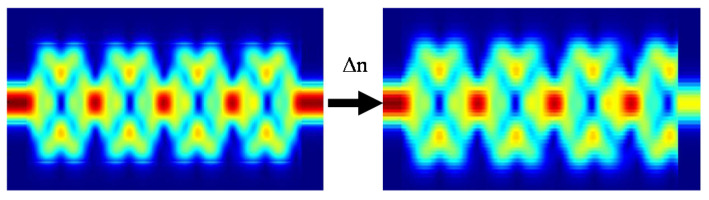
Visualizing the effect of Δn = 0.3. (**Left**) Background index 1.3. 4 self-images in a multimode section with length 94.1 μm for the background refractive index 1.3. (**Right**) Background index 1.6. The beating length increased due to the increased n_eff_, and thus, taking the output at the same distance of 94.1 μm results in much lower transmission.

Figure 2 can be used to explain the mechanism of the MMI device as a sensor. The device on the left has maximum transmission as it is designed for self-imaging to occur at that length. Taking a measurement at the same operating frequency but with a different refractive index of analyte results in much lower transmission. A spectral response can identify that the maximum transmission occurs at a different wavelength due to the change in refractive index. Therefore, there is a measurable spectral shift that depends on the refractive index of the analyte.

**Figure 3:**
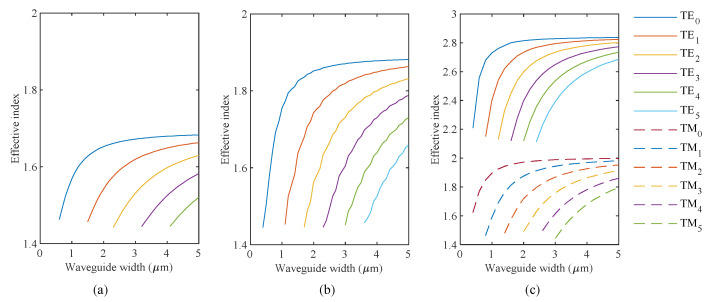
Effective indices of guided modes for various widths for (**a**) 50 nm height, (**b**) 70 nm height, and (**c**) 220 nm height waveguides.

Before designing the MMI devices, it is useful to review the dispersion analysis of SOI waveguides of varying heights, shown in Figure 3a–c. 

Fifty nm and 70 nm ultrathin waveguides support only transverse electric (TE) modes. The analytical solution for a transverse magnetic TM mode of silicon slab waveguide on silicon dioxide substrate results in effective refractive indices of 1.463 and 1.475 for the 50 nm and 70 nm slab thickness, respectively. These numbers are close to the refractive index of SiO_2_, which is why these ultrathin waveguides do not support a TM mode. The ultrathin 50 nm waveguide platform could be fabricated using a standard foundry process supporting partial etch. An example would be a silicon photonics process starting with a SOI wafer with a BOX layer of 2 microns, 220 nm device layer, and 150 nm partial etch, leaving behind 70 nm.

For the 220 nm thick waveguides, we analyzed a width that results in three modes (w = 1.4 μm), and another that results in four modes (w = 1.8 μm). As explained previously [33], using a multimode section supporting more than five modes increases losses with no additional improvements in sensitivity. Thus, for the 50 nm and 70 nm ultrathin waveguides, we fixed the multimode section width to 4.5 μm and 3 μm, respectively. The analysis in Figure 3 shows that these dimensions support five modes. Equation (2) describes the self-imaging length for symmetrical injection as in our case, where *p* is an integer.

(2) $$L=p((3L_{\pi })/4)$$

The longer the MMI section, the more light-matter interaction there is. Several MMI section lengths corresponding to different multiples of the self-imaging length were analyzed for each of the waveguides. A commercial simulator eigenmode solver was used to perform the calculations [34].

### 2.2. Experimental—Fabrication and Characterization

The validation of the simulation method was performed by comparing the simulation results with the experimental results for the devices based on the 220 nm device layer platform. Although the rational for this work was that the fabrication doesn’t require advanced lithographical techniques, we used E-beam lithography as a prototyping method but the geometries used, were compatible with the UV lithography. The fabrication was part of a multi-project wafer (MPW) run where all waveguides were covered with oxide cladding; therefore, the fabricated MMI devices’ spectral responses were only characterized experimentally with an oxide cladding.

Fabrication was through Applied Nanotools Inc. using their NanoSOI MPW, employing direct-write 100 keV electron beam lithography technology (http://www.appliednt.com/nanosoi; Edmonton, AB, Canada). The substrates were 8-inch silicon-on-insulator (SOI) wafers with 2 µm thick buffer oxide and 220 nm thick device layer. Hydrogen silsesquioxane was deposited by spin-coating followed by baking. A Raith EBPG 5000+ electron beam instrument with raster step size of 5 nm was used to pattern devices followed by development with a tetramethylammonium sulfate solution. Etching was performed by inductively coupled plasma reactive ion etching (ICP-RIE) using chlorine gas then remaining resist was removed in 10:1 buffer oxide wet etch. A plasma enhanced chemical vapor deposition (PECVD) process using tetraethyl orthosilicate (TEOS) at 300 °C was used to deposit oxide cladding of 2.2 µm thickness.

For characterization, an automated probe station (Maple Leaf Photonics) was used to test the fabricated 220 nm height devices using a fiber array on a temperature-controlled stage set at 25 °C. To allow a comparison with the simulations, a loopback structure consisting of input and output fiber grating couplers connected with a straight single-mode waveguide was used to capture the spectral response of the fiber-grating couplers and associated losses.

## 3. Results

### 3.1. Experimental Results of the MMI Devices Based on the 220 nm Device Layer Platform

**Figure 4:**
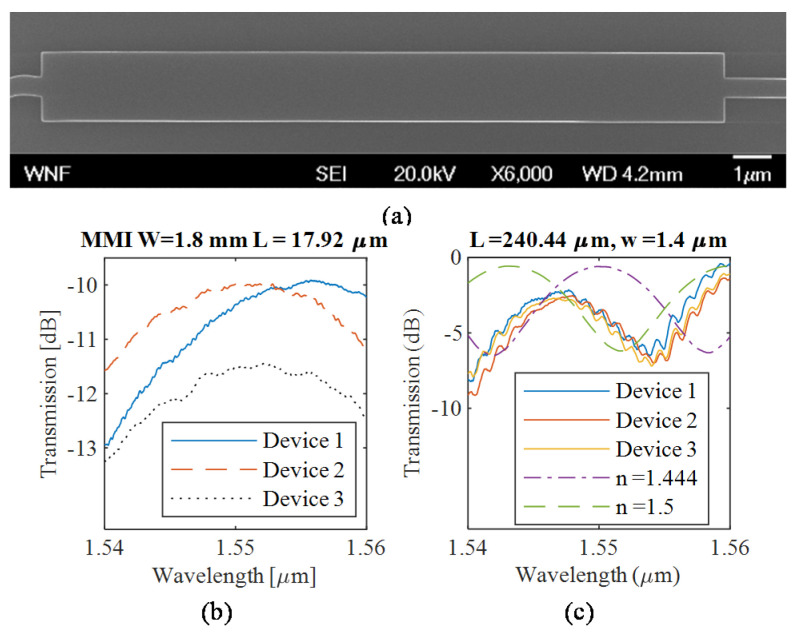
(**a**) SEM image of MMI device fabricated by electron beam lithography using a 220 nm device layer platform with 1.8 μm width and 17.92 μm length. (**b**) Raw transmission spectra of three replicas of the MMI device fabricated in different areas of the chip. (**c**) Comparison of the simulation and experimental results after loopback compensation for an MMI with width 1.4 μm and length 240.44 μm. Shown in the dash-dotted line is the original simulation using n = 1.444 as the oxide layer refractive index, shown in the dashed line is a re-simulation using n = 1.5 as the oxide layer refractive index, which is closer to the experimental results.

This work is mainly simulation work. Nevertheless, we include here the experimental analysis of the MMI devices with height h = 220 nm that are similar to the devices that are designed in this paper with height h = 50 nm or 70 nm as verification of our simulation methodology. MMI devices with height h = 220 nm were first analyzed. Fully etched sub-wavelength fiber grating couplers [35] were used to couple light to the devices. Figure 4a shows a sample device SEM. Figure 4b shows the raw output of three replicas. A loopback structure was fabricated to be able to remove the spectral response of the grating couplers to enable comparison with simulations. Figure 4c shows spectra after compensating for the spectral response of the grating couplers and compares the experimental and simulation results. We attribute the discrepancy to the fabrication error. There is a ~200 nm difference in width between the simulated and fabricated MMI in Figure 4a. Reviewing Equation (1), it is expected that small variations in fabrication lead to significant changes in the response due to the $w_{e}^{2}$ term. This discrepancy is not expected to be problematic in a real sensor application. Such variations could be accounted for by proper device calibration, i.e., the spectral response with liquids of known refractive index to be measured prior to performing the actual measurements.

### 3.2. Simulation Results of Ultrathin MMI Sensors

**Figure 5:**
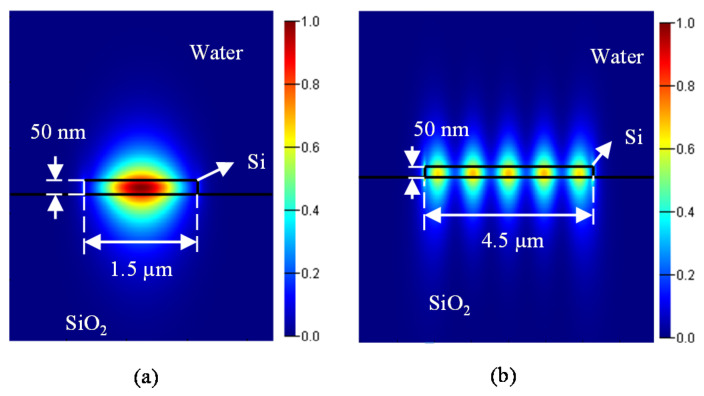
(**a**) Mode profile of single mode waveguide, (**b**) mode profile of multimode section.

The guided mode profiles of the input waveguide and the multimode section of the h = 50 nm waveguide is shown in Figure 5a,b.

As explained earlier, these ultrathin waveguides do not support guided TM modes. However, the fiber might inadvertently couple TM modes to the single-mode input waveguide, so it is important to ensure that the distance between the grating coupler and the multimode section is larger than the TM mode’s propagation distance. The propagation distance of the TM mode for the 1.5 µm wide input waveguide with h = 50 nm is 16 µm, and the propagation distance for the 1 µm wide input waveguide with h = 70 nm is 70 µm.

**Figure 6:**
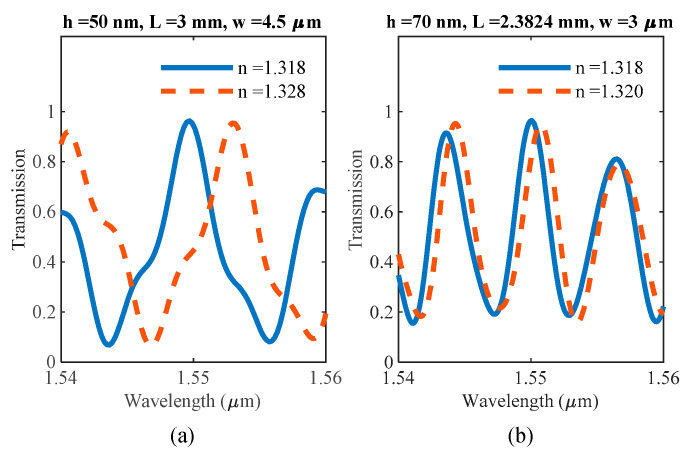
Spectral response of the sensor with reference (blue-solid line) taken as water with n = 1.318. Peak transmission due to self-imaging is at 1550 nm wavelength. The red dashed line shows the red-shift due to a change in the refractive index. (**a**) silicon device layer thickness 50 nm, MMI section width 4.5 μm, MMI section length 3 mm, Δ*n* = 0.01. (**b**) silicon device layer thickness 70 nm, MMI section width 3 μm, MMI section length 2.4 mm, Δ*n* = 0.002.

The spectral responses of ultrathin sensors with h = 50 nm and h = 70 nm are shown in Figure 6. The lengths of the sensors were varied to determine how various performance metrics change with device length, and the ultrathin MMI sensors are compared to the 220 nm thick sensors, as shown in Figure 6. All the lengths used follow Equation (2) such that self-imaging occurs at these lengths with 1550 nm wavelength.

**Figure 7:**
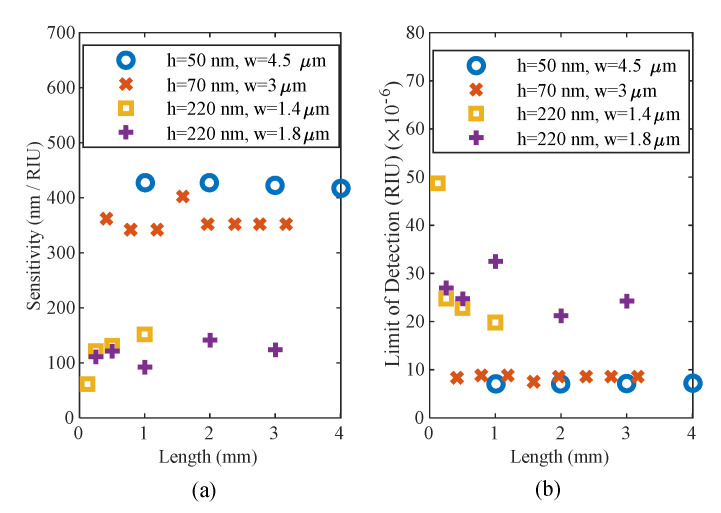
(**a**) Sensitivity and (**b**) limit of detection for MMI sensors of different geometries.

The spectral shift due to a change in the refractive index is used as a performance indicator of the sensor, i.e., the sensitivity as in (3) and shown in Figure 7a:

(3) Sensitivity=Δλ/Δn (nm/RIU)

Limit of detection (LOD) (4) is linked to the sensitivity S and the noise level ϵ [36] and can be estimated using:

(4) LOD=3ε/S (RIU)

Using a noise level estimate of 1 pm [36], the LOD of the MMI sensors are reported in Figure 7b. As expected, the thinnest 50 nm MMI is the most sensitive due to the largest leakage of the mode out of the core, thus being more strongly affected by the surrounding medium. The 50 nm ultrathin MMI demonstrated a sensitivity of ~420 nm/RIU while the 70 nm thick MMI sensitivity was calculated to be ~350 nm/RIU.

The full-width at half-maximum (FWHM) and free spectral range (FSR) are strongly dependent on the MMI section length. We defined the figure of merit (FOM) for our sensors by (5):

*Figure of merit* = *Sensitivity*/*FWHM* (5)

A narrower full width at half maximum (FWHM) makes it easier to extricate the spectral shift, and thus this definition of the FOM is experimentally useful. The free spectral range’s FSR significance is in the maximum Δn that could be imposed. A Δn causing a Δλ larger than the FSR will give an ambiguous spectral shift and the results will be difficult to interpret. 

**Figure 8:**
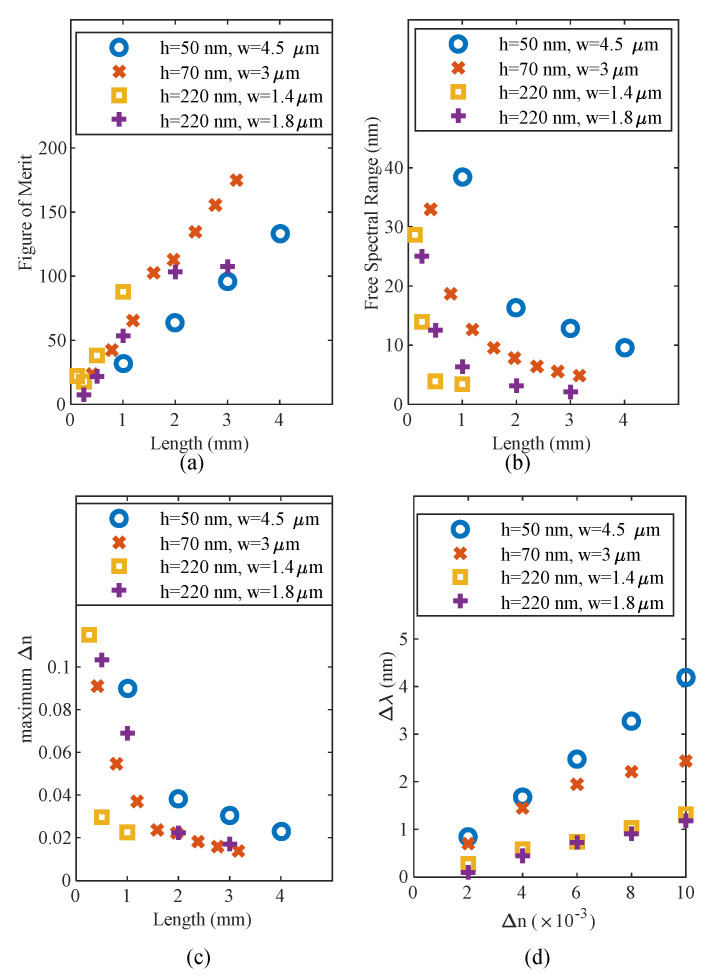
Performance metrics for the MMI sensor based on the ultrathin waveguide platforms: 50 nm (blue circles) and the 70 nm (red crosses) silicon device layer. (**a**) Figure of merit as defined in (5) indicates the ease of measuring a spectral shift. (**b**) Free spectral range is reduced with increasing length, causing a reduction in the maximum discernible Δn, (**c**). (**d**) Wavelength shift versus refractive index change of the bulk medium. Lengths used in this simulation: blue circles: 4.007 mm, red crosses: 2.382 mm, yellow squares:1 mm, purple plus signs: 1.003 mm. Operating wavelength 1550 nm.

Figure 8a compares the FOM for the different sensor platforms. Despite the 50 nm thick platform providing a better sensitivity than the 70 nm platform, the 70 nm platform provides sharper transmission peaks, and thus, outperforms the 50 nm platform using the FOM defined in (5). Figure 8b,c shows that the longer MMI section length corresponds to a narrower FSR, and thus, a smaller discernable Δn. Figure 8d demonstrates the linearity of the sensors.

### 3.3. Applications of Ultrathin MMI Sensors

**Figure 9:**
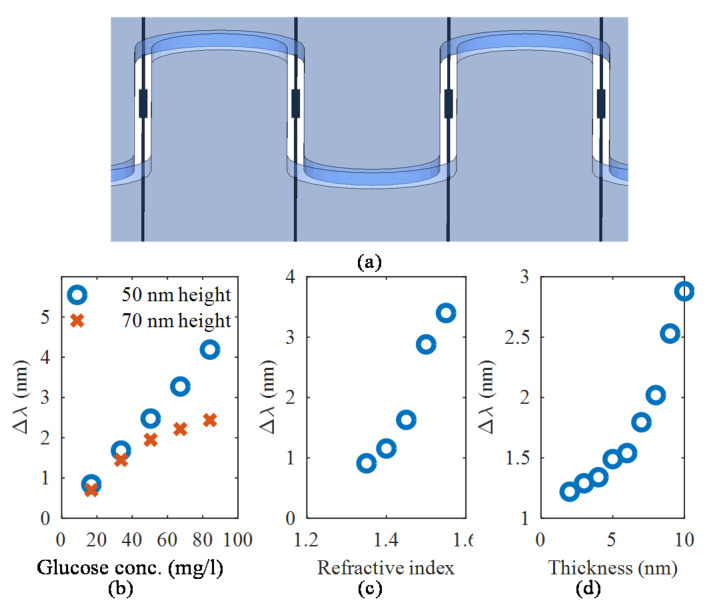
(**a**) Each MMI sensor functionalized for multiplex sensing. Sensor performance for (**b**) bulk glucose sensing, (**c**) protein sensing using a protein layer of 10 nm thickness with varying refractive indices using the 70 nm thick device, and (**d**) protein sensing using a biotin-streptavidin protein layer (*n* = 1.502) of varying thicknesses using the 70 nm device.

The MMI sensors are suitable for a wide variety of applications. Multiple MMI biosensors, each functionalized for a different application, can be integrated on-a-chip with microfluidics channels, as shown in Figure 9a. For the following analysis, only the ultrathin MMI devices were analyzed.

For glucose sensing, the bulk sensitivity was most suitable, as has already been explored in Section 3.2. The refractive index of glucose varies as a function of its concentration C [36], as in (6):

(6) $$n_{glu\cos e}=aC+b$$

where C is measured in g L^−1^, a is 1.189 × 10^−4^ [36,37], and b is the refractive index of water at 1550 nm wavelength, which we have taken to be 1.318 following [38]. Based on this information, the refractive indices of the various concentrations of glucose solutions were calculated and the corresponding peak shift simulated using the length of 2.4 mm for the 70 nm device layer platform and 4 mm for the 50 nm device layer platform. Figure 9b shows how much the peak shifts for different glucose concentrations where it could be observed that the MMI sensor with the 50 nm device layer is linear for a wider range of glucose concentrations, and it is more sensitive than the 70 nm thick device. The limits of detection are 6 mg dL^−1^ and 7.2 mg dL^−1^ for the 50 nm and 70 device layer MMI sensors, respectively. Critical values related to diabetes are around 100 to 200 mg dL^−1^ [39].

We also explored the suitability of the MMI sensor for surface sensitivity. In many biosensing applications, molecules such as proteins attach to the surface of the sensor. In this study, we used only the sensor with 70 nm thick device layer. The higher the protein concentration, the thicker the layer on top of the sensor. To assess the suitability of the sensor for a wide variety of proteins, we used a fixed protein layer thickness of 10 nm and varied the refractive index from 1.3 to 1.6, covering a variety of proteins such as bovine serum albumin (n = 1.47 [40]) and biotin-streptavidin (n = 1.502 [41]), and the results are summarized in Figure 9c. As a further study, we focused on biotin-strepatividin and studied the effect of the protein layer thickness and the results are summarized in Figure 9d.

## 4. Discussion

Due to our judicious choice of the SOI platform and the high index contrast between SiO_2_ and Si, the sensor is much more compact than previous MMI sensors. Table 1 compares our sensor with MMI sensors in the literature. Despite the 50 nm device layer sensor being more sensitive and having a lower limit of detection, there are several fabs that have optimized processes to work with the 70 nm thickness, and there are commercially available SOI wafers with 70 nm thick device layer.

## 5. Conclusions

The effects of the waveguide width on the number of guided modes were presented for 50 nm and 70 nm thick SOI waveguides. This analysis allowed the optimization of MMI sensor designs based on these platforms. Such ultrathin silicon device layers are realizable in standard silicon photonics runs that include a partial etch step. Particular applications will require the designer to understand the tradeoffs. At a length of 4 mm, the 50 nm silicon device layer platform provides a sensitivity of 420 nm/RIU, FOM of 133, and a maximum Δ*n* of 0.023. On the other hand, the 70 nm silicon device layer platform results in a more compact design: only 2.4 mm length was required to achieve similar FOM of 134 while having maximum Δ*n* of 0.018. The ultrathin SOI waveguide platform is promising in sensing applications using this simple MMI structure, which can easily be integrated with microfluidics components.

## Figures and Tables

**Table 1:** Comparison of this work with state of the art MMI sensors.

Work	Sensitivity	Footprint	Study
This work—50 nm	420 nm/RIU	4.5 μm × 4 mm	Sim
This work—70 nm	350 nm/RIU	3 μm × 2.4 mm	Sim
Si-on-Si [27]	0.8/°C	32 μm × 5 mm	Sim
Doped silica [25]	1900 nm/RIU	7 μm × 30 mm	Sim
Multimode fiber [26]	297 nm/RIU	125 μm × 58.6 mm	Exp
SOI [42]	0.0364 THz/°C	27 μm × 1.144 mm	Sim
